# Assessing the Utility of Hemoglobin, HALP Score, FAR Ratio, and Coagulation Parameters as Predictors for Preterm Birth

**DOI:** 10.3390/children10030527

**Published:** 2023-03-08

**Authors:** Ingrid Hrubaru, Andrei Motoc, Catalin Dumitru, Felix Bratosin, Roxana Manuela Fericean, Satish Alambaram, Ioana Mihaela Citu, Gratiana Nicoleta Chicin, Izabella Erdelean, Florin Gorun, Cosmin Citu, Zoran Laurentiu Popa

**Affiliations:** 1 Department of Obstetrics and Gynecology, “Victor Babes” University of Medicine and Pharmacy Timisoara, Eftimie Murgu Square 2, 300041 Timisoara, Romania; 2 Doctoral School, “Victor Babes” University of Medicine and Pharmacy Timisoara, Eftimie Murgu Square 2, 300041 Timisoara, Romania; 3 Department of Anatomy and Embryology, “Victor Babes” University of Medicine and Pharmacy Timisoara, Eftimie Murgu Square 2, 300041 Timisoara, Romania; 4 Department XIII, Discipline of Infectious Diseases, University of Medicine and Pharmacy “Victor Babes” Timisoara, Eftimie Murgu Square 2, 300041 Timisoara, Romania; 5 Bhaskar Medical College, Amdapur Road 156-162, Hyderabad 500075, India; 6 Department of Internal Medicine I, “Victor Babes” University of Medicine and Pharmacy Timisoara, Eftimie Murgu Square 2, 300041 Timisoara, Romania; 7 Faculty of General Medicine, “Vasile Goldis” Western University of Arad, Bulevardul Revolutiei 94, 310025 Arad, Romania; 8 National Institute of Public Health, Strada Doctor Leonte Anastasievici 1-3, 050463 Bucuresti, Romania

**Keywords:** preterm delivery, preterm labor, premature birth, predictive factors, inflammation, anemia

## Abstract

Premature birth is a worldwide health issue, posing a high mortality risk for newborns, as well as causing emotional and financial difficulties, and long-term health issues for patients. Identifying effective predictors for preterm birth is essential for prolonging gestation or improving obstetric care. As invasive methods are costly, risky, and not universally available, we aim to assess the predictive capacity of various serum parameters in pregnant women during the third trimester, as a non-invasive alternative. Based on previous studies, it was hypothesized that hemoglobin, the association of hemoglobin, albumin, lymphocyte, and platelets’ (HALP) score, and coagulation parameters such as the prothrombin time (PT), activated partial thromboplastin clotting time (aPTT), D-dimers, and fibrinogen to albumin ratio (FAR) have significant prediction capabilities. With a retrospective design, a total of 161 patients with a history of preterm birth were included in the analysis, being matched 1:1 with a control group of women who gave birth at term. All laboratory samples were collected during the third trimester of pregnancy. The computed area under the curve (AUC) ranged between 0.600 and 0.700 in all six studied parameters, suggesting a fair discrimination. The highest predictive value for preterm birth was observed to be represented by the HALP score with AUC = 0.680 and the highest sensitivity (75%, *p*-value = 0.001). The highest specificity was achieved by the prothrombin time (69%), and the HALP score was also 69%. The FAR score had an AUC of 0.646, with a sensitivity of 68%, and specificity of 64% (*p*-value = 0.020). All other variables were significant estimates for the risk of preterm birth, although with lower accuracy. Pregnant women with a hemoglobin level below 12.0 g/dL had a 3.28 higher likelihood of giving birth prematurely. A prothrombin time below 12.5 s determined a 2.11 times higher risk of preterm birth. Similarly, the aPTT below 25 s was linked with 3.24 higher odds of giving birth prematurely. However, the strongest predictors were the D-dimers above 250 ng/mL (OR = 4.26), the FAR score below 0.1, with an odds ratio of 5.30, and the HALP score with a 6.09 OR for a cut-off value above 24. It is important to determine these parameters in pregnant women at risk for giving birth prematurely, but further external validation is required to confirm these findings.

## 1. Introduction

Premature birth, which is considered as giving birth before 37 weeks of pregnancy, is a global issue of the maximum importance [1]. It impacts more than 5% of all births worldwide and constitutes a tremendous burden on healthcare systems [2]. Premature birth is the leading causative factor of infant and neonatal death [3]. In addition to its impact on child mortality, there is growing proof that newborns born prematurely are at a higher risk of having certain prematurity-related disorders, such as developmental impairments and neurologic dysfunction [4,5]. Preterm birth has also been related to an elevated incidence of several adult chronic conditions, including cardiovascular diseases, type 2 diabetes, and mental health problems [6].

Increasing data reveal that preterm birth is a condition caused by the interaction of various variables, including heredity, inflammation, disturbance of maternal hormones, placental vascular abnormalities, and numerous more hypotheses [7,8,9]. Due to the complex etiology, previous research has shown that precise forecasts are still challenging. In the majority of healthcare settings, the prediction of preterm birth is still largely based on subjective medical knowledge [10,11]. Such an approach may increase both unneeded hospital admissions and inappropriate interventions with the possibility of causing injury to the maternal–fetal dyad [12,13].

So far, the use of non-invasive methods to determine the risk of premature birth was established on the accuracy of serum parameters responsible for anemia and different scoring methods based on the inflammatory status of the pregnant woman, such as the Neutrophil–Lymphocyte Ratio, Monocyte-to-Lymphocyte Ratio, Platelets-to-Lymphocyte Ratio, Systemic immune–inflammation index, or the systemic inflammatory response index [14,15,16]. Therefore, the aim of this retrospective research is to determine the prognostic value of hemoglobin, the association of hemoglobin, albumin, lymphocyte, and platelets’ (HALP) score, and coagulation parameters such as the prothrombin time (PT), activated partial thromboplastin clotting time (aPTT), D-dimers, and fibrinogen to albumin ratio (FAR) for the risk of premature birth.

## 2. Materials and Methods

### 2.1. Study Design and Ethical Considerations

The current study was designed as a retrospective cohort at the Clinic of Obstetrics and Gynecology “Odobescu” from Timisoara, Romania, in affiliation with the Victor Babes University of Medicine and Pharmacy and the Emergency Municipal Clinical Hospital from Timisoara, Romania. The research design and methodology were approved by the two affiliated institutions to collect, analyze, and publish patient data from 1 January 2020 until 31 December 2021, in accordance with the guidelines of the Declaration of Helsinki, with the approval number E-2814 from 19 May 2022.

### 2.2. Inclusion and Exclusion Criteria

**Figure 1:**
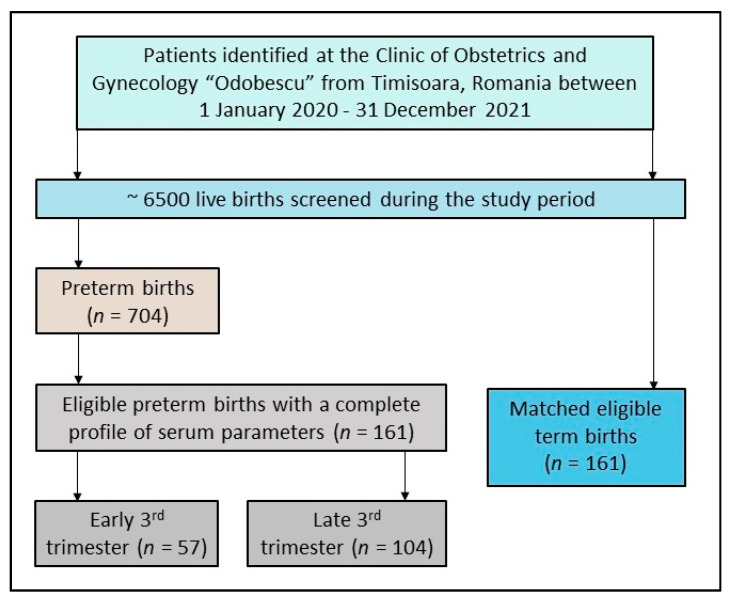
Patient selection flowchart.

Patients considered for inclusion were pregnant women identified in the hospital database during the aforementioned study period. The inclusion criteria comprised the following: (1)—history of preterm birth; (2)—age over 18 years; (3)—consent for personal data utilization; (4)—complete medical records; (5)—all laboratory samples were measured during the third trimester of pregnancy. Among the third-trimester measurements in the prematurity group, one-third were in the early third trimester (between 28 and 31 weeks of gestation), and the other two-thirds were measured in the late third trimester, between 32 and 36 weeks of gestation. Patients were excluded from this study based on the following criteria: (1)—being underage; (2)—missing consent for private data utilization; (3)—incomplete medical records regarding the analysis of a complete blood count, albumin, and the coagulation parameters (PT, aPTT, D-dimers, and fibrinogen); (4)—laboratory data from the first- or second-trimester of pregnancy. To avoid confounding factors, patients were also excluded if they had a diagnosis of coagulation disorders, or infections and inflammatory illnesses. Other exclusion criteria were the use of anti-inflammatory and corticosteroid medications, which might have affected the serum parameters during pregnancy. A control group of pregnant women without a history of preterm birth was collected and was matched with the patients from the preterm birth group based on age, number of gestations, and number of comorbidities at a 1-to-1 ratio, as described in Figure 1. The comorbidities that were used for matching included the following conditions identified before the actual pregnancy: diabetes mellitus, thyroid disorders, high blood pressure, depression, urinary tract infections, anemia, and asthma.

According to existing guidelines, preterm delivery was considered when the birth happened before 37 weeks of gestation [17]. The third trimester of pregnancy was considered as the period between 27 weeks of gestation until birth [18,19], and all laboratory data presented in this study were collected during the third trimester, before the onset of labor. The HALP score was computed based on the formula hemoglobin (g/L) × albumin (g/L) × lymphocytes (/L)/platelets (/L) [20].

### 2.3. Variables

The variables proposed for analysis in the current study included background characteristics of the pregnant women (age, body mass index, gravidity, parity, number of comorbidities, substance use during pregnancy, obstetrical characteristics) and serum parameters (white blood cell count, lymphocytes, neutrophils, platelets, hemoglobin, albumin, prothrombin time, activated partial thromboplastin clotting time, d-dimers, fibrinogen, HALP score, and FAR score).

### 2.4. Statistical Analysis

The sample size was determined using a convenience sampling method. It was calculated that 128 cases represent the ideal sample size, considering a rate of premature births in the general population of up to 10%, a margin of error of 5%, and a confidence level of 99%. The threshold for statistical significance was 0.05. The statistical power (1-β) calculation was 80%, for a type I error rate of 5%. Data were sorted by variables based on the type of their distribution and evaluated with the Kolmogorov–Smirnov test. Using the Student’s *t*-test, the mean and standard deviation of Gaussian variables were compared. The proportions of categorical variables were compared with the Chi^2^ test. The chance of preterm was analyzed and described by a Kaplan–Meier curve. To determine the risk of preterm delivery, a multiple linear regression analysis was conducted, that was adjusted for confounding factors. The risk of preterm birth prediction performance was examined using the receiver operating characteristic (ROC) curve method by calculating the area under the curve (AUC) and its significant related value. The ideal cut-off values for inflammatory indices were determined using Youden’s index.

## 3. Results

### 3.1. Patients’ Background

At the end of the patient selection process, a total of 322 eligible pregnant women were included in the analysis with a complete background profile and laboratory profile. Table 1 describes the background characteristics of the patients analyzed in the study stratified by the status of premature birth. Since the two study groups were matched by age, the number of gestations, and the number of comorbidities, there were no significant differences. The average age of patients in the prematurity group was 29.4 years and 29.6 years in the “no prematurity” group, respectively. There was a statistically significant difference between the average value of the body mass index between women who gave birth prematurely and those who did not (26.3 kg/m^2^ vs. 24.3 kg/m^2^, *p*-value < 0.001). Another significant difference was the proportion of smokers, since 11.2% of women in the prematurity group were smoking during pregnancy, compared to 5.0% in the control group (*p*-value = 0.040).

Regarding the obstetrical characteristics, it was observed that premature rupture of membranes occurred significantly more often in the prematurity group (10.6% vs. 2.5%, *p*-value = 0.003). The onset of preterm labor was at 35.3 weeks in the prematurity group, while the term labor occurred at around 37.9 weeks of gestation in the control group (*p*-value < 0.001). On the other side, there were significantly more c-sections performed in the control group (20.5% vs. 10.6%, *p*-value = 0.013). It was also observed that pregnant women in the prematurity group suffered from urinary tract infections significantly more often than patients in the other group (25.5% vs. 16.1%, *p*-value = 0.039).

### 3.2. Serum Parameters

The serum parameters analyzed to assess the risk of preterm birth are presented in Table 2. It was observed that there was a statistically significant difference between the mean values of lymphocytes in the prematurity and “no prematurity” groups since women who gave birth prematurely had a lower lymphocyte count than the control group (0.78 × 10^9^/L vs. 1.06 × 10^9^/L, *p*-value < 0.001). Additionally, hemoglobin levels were significantly decreased among women from the prematurity group (113 g/L vs. 139 g/L, *p*-value < 0.001). The coagulation parameters PT and aPTT were also decreased in the prematurity group (12.9 s vs. 13.3 s, *p*-value = 0.041) and 26.7 s vs. 27.9 s (*p*-value < 0.001), respectively. The average albumin levels were 31.4 g/L among women who gave birth prematurely, compared with 36.6 g/L among those who gave birth at term (*p*-value < 0.001). Fibrinogen and d-dimers, on the other hand, were significantly more elevated in the prematurity group (229 ng/mL vs. 216 ng/mL, *p*-value = 0.009) and 5.13 g/L vs. 3.10 g/L in the “no prematurity” group (*p*-value < 0.001), respectively. The mean HALP score value among women who gave birth prematurely was 12.82, compared to 23.96 in the control group (*p*-value < 0.001). The FAR score was above the threshold in the prematurity group (0.16 vs. 0.08, *p*-value < 0.001).

### 3.3. Receiver Operand Curve and Area under Curve

**Figure 2:**
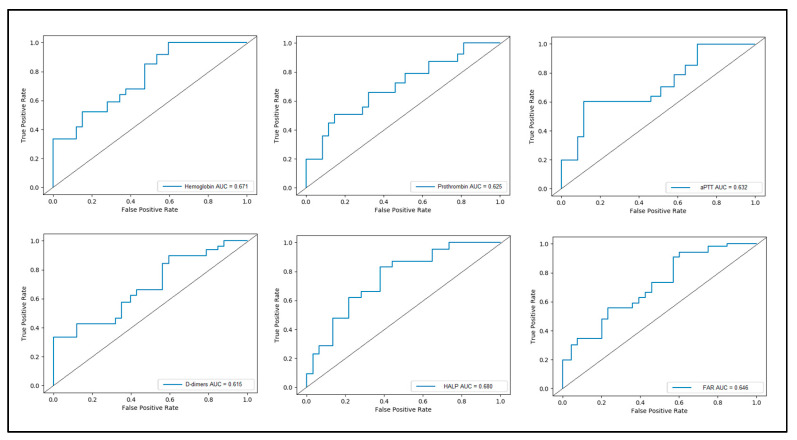
ROC plot for the variables involved in estimating the risk for preterm birth.

Table 3 and Figure 2 present the receiver operating characteristic plot for the variables involved in estimating the risk for preterm birth. The computed area under the curve (AUC) was above 0.600 in all six studied parameters, although none of them was higher than 0.700. Therefore, it can be considered that they have a poor discrimination, even though they have statistical significance. The highest value was observed to be represented by the HALP score with AUC = 0.680 and the highest sensitivity as well (75%, *p*-value = 0.001). The highest specificity was achieved by the prothrombin time (69%), and the HALP score was also 69%. The FAR score had an AUC of 0.646, with a sensitivity of 68% and specificity of 64% (*p*-value = 0.020). All other variables were significant estimates for the risk of preterm birth, although with lower accuracy.

### 3.4. Risk Assessment

**Figure 3:**
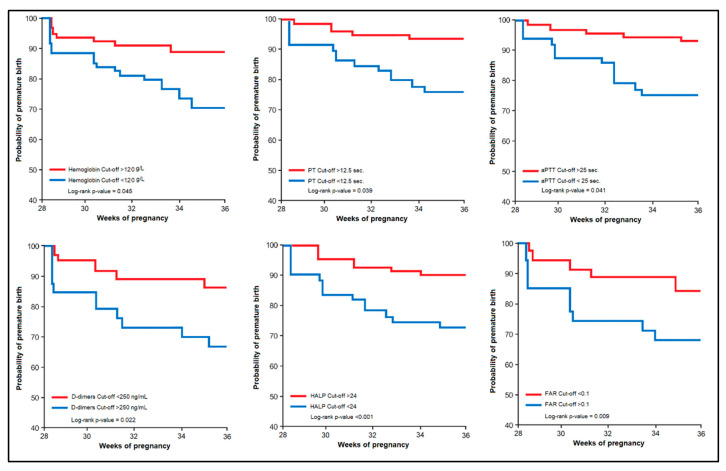
Kaplan–Meier plot for the estimated probability of preterm birth.

**Figure 4:**
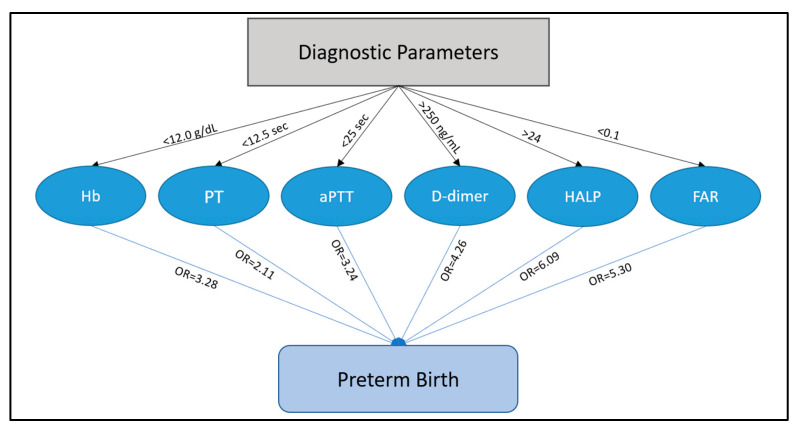
Flowchart of factors associated with preterm birth.

The risk assessment for preterm birth based on maternal serum parameters is presented in Table 4 as the regression analysis, while Figure 3 and Figure 4 describe the threshold for significance in premature birth assessment based on a Kaplan–Meier plot. Therefore, pregnant women with hemoglobin levels below 120 g/L (12.0 g/dL) had a 3.28 higher likelihood of giving birth prematurely (*p*-value < 0.001). A prothrombin time below 12.5 s determined a 2.11 times higher risk of preterm birth (*p*-value = 0.038). Similarly, the aPTT below 25 s was linked with 3.24 higher odds of giving birth prematurely. However, the strongest predictors were the D-dimers above 250 ng/mL (OR = 4.26), the FAR score below 0.1, with an odds ratio of 5.30, and the HALP score with a 6.09 OR for a cut-off value above 24 (*p*-value < 0.001).

## 4. Discussion

### 4.1. Important Findings

The presented diagnosis scores might have the potential to be applied in clinical practice to prevent premature birth. Invasive methods for predicting preterm birth are costly, risky, and not universally available, and thus, identifying effective non-invasive predictors is crucial. The parameters assessed in this study, including hemoglobin, HALP score, and coagulation parameters, could be measured during routine prenatal care visits and may offer a non-invasive method for identifying women at high risk of preterm birth. Another possible application is to monitor women who are already at high risk of preterm birth due to underlying medical conditions or previous history. Regular measurement of these parameters during prenatal care visits may allow for the earlier detection of changes in these scores, indicating an increased risk of preterm birth. This may prompt healthcare providers to offer closer monitoring or earlier intervention to prevent preterm birth. However, it is important to note that further external validation is required to confirm the findings of this study and determine the optimal cutoff values for each parameter. Additionally, cost-effectiveness and accessibility of these non-invasive methods in different healthcare settings need to be considered before implementing these diagnosis scores in clinical practice.

The present research found that hemoglobin levels below 12.0 g/dL, prothrombin time below 12.5 s, aPTT below 25 s, D-dimers over 250 ng/mL, a FAR score below 0.1, and a HALP score above 24 were significant predictors of preterm delivery in the third trimester. In this context, values that fall above the cut-off values are statistically insignificant predictors for preterm birth. The HALP score also had the highest sensitivity for preterm birth, with 75%, and a likelihood of 6.09 times higher than the reference range. However, the HALP score has so far mostly been investigated in correlation with malignancies. One study found that the association of hemoglobin, albumin, lymphocyte levels, and platelets represents a good predictor for negative outcomes in patients with cancer [21]. A HALP score of less than 22 was related to a more advanced stage and bigger tumor size, consistent with studies of other malignancies in which at least one characteristic of a more advanced stage or greater tumor size was present in the low HALP group [22,23]. A HALP score below 22 was independently related to lower progression-free survival and overall survival from the perspective of mortality. This conclusion was consistent with the findings of numerous previous studies that revealed a correlation between low HALP and a worse prognosis for cancer patients.

Multiple processes may describe the effects of inflammation and dietary status on the HALP score and preterm birth. A greater proinflammatory condition reduces erythrocyte survival, inhibits bone marrow activity, and induces hypoferremia, leading to decreased hemoglobin concentration. Moreover, low albumin levels, a component of the HALP score, have been described during pregnancy as accounting for adverse events. In pregnancy-related hypertension, serum albumin level is a strong predictor of illness severity and may be regarded as a valuable measure for predicting time to delivery, proteinuria severity, and pregnancy outcomes [24]. In addition, it has been shown that low blood albumin concentrations increase the formation of oxygen-derived free radicals, which in turn compromise the vascular endothelial function of the mother, which may lead to adverse pregnancy outcomes such as premature delivery [25].

One of the strongest predictors identified during the third trimester for preterm birth was hemoglobin. We identified that anemia with a hemoglobin level below 120 g/L was associated with a 3.28 higher likelihood of premature delivery. One meta-analysis observed that there was no significant correlation between maternal anemia during pregnancy and preterm delivery [26]. The research revealed that maternal anemia with hemoglobin levels below 11 g/dL during the first trimester increased the risk of preterm delivery by a factor of 1.26. However, this association was not significant throughout the second and third trimesters. The scientists also demonstrated that the correlation between maternal anemia during pregnancy and rising preterm birth rates is more prevalent in developing nations.

Although our study described a high proportion of pregnant women with anemia, the type of anemia was not investigated, even though different types of anemia might be stronger determinants for preterm labor. In another research study, the anemia was primarily of the normocytic normochromic variation with a prevalence rate of more than 50% [27]. Similar results were reported earlier, showing significant normocytic normochromic anemia among pregnant women who gave birth prematurely [28]. Another study came to the conclusion that young mothers are more likely to experience gestational anemia [29]. However, these findings do not comply with our findings since we did not attempt to include the age of patients as a determining factor for complications that can lead to giving birth prematurely. In addition, several research studies have shown the impact of anemia on neonates, who were delivered with a low APGAR score at 1 min from anemic females [30]. These findings suggested that there is a two-fold rise in the occurrence of a low APGAR score in anemic mothers compared to non-anemic women. The incidence of small for gestational age in anemic mothers was three to five times higher in the anemic population compared to non-anemic population [31], although this conclusion is inconsistent with other research that says that there is no association between maternal anemia and the diagnosis of small for gestational age [32]. Therefore, different ranges of hemoglobin will affect the maternal outcome in weight, the APGAR score, and other anthropometric indices of the infant.

Maternal anemia is a risk factor for adverse pregnancy outcomes and poses a danger to the fetus’s survival. The preponderance of fetal and maternal problems, including abortions, obstructed labor, postpartum hemorrhage, and pre-eclampsia, were found in anemic women [33]. Another recent research study revealed a correlation between anemia severity and the risk of preterm birth [34]. In our research, neither the assessment of hemoglobin levels in the first and second trimesters nor the estimation of average values were conducted. Hemoglobin readings in many research studies were most likely taken in the late second or third trimester, since the hemoglobin levels of pregnant women decline during the course of the pregnancy and reach their lowest levels in the late third trimester. Another noteworthy piece of research disregarded third-trimester hemoglobin readings because dilutional anemia, which occurs as a 50% increase in plasma volume during this time, is not compensated for by an increase in the number of red blood cells in the late third trimester of pregnancy [27]. The same research identified the impact of anemia on preterm delivery as determining a 2.42 times higher risk when the hemoglobin levels were below 10 mg/dL and 1.77 times higher when the hemoglobin levels were below 11 mg/dL, respectively.

Earlier research indicated that hypercoagulation might be linked to premature delivery. In agreement with our results, additional research studies found substantially shorter PT and aPTT in women with PUC who had preterm labor compared to those with normal term birth, indicating that both the intrinsic and extrinsic processes are active in preterm birth [35]. Shorter PT and aPTT are universal coagulation assays used to evaluate the coagulation system and have been identified as risk factors for venous thromboembolism in the past. One of the probable reasons for the shorter aPTT reported in women with preterm labor might be the slightly increased levels of Von Willebrand Factor antigen and factor VIII activity observed in this group. In addition, higher levels of coagulation factors such as factor VII, factor XI, and factor IX, which were not examined in our research, are likely to have contributed to the observed decrease in PT and aPTT.

Other studies propose the evaluation of coagulation parameters such as PT and aPTT for the risk of preterm birth, but also for the risk of intraventricular hemorrhage [36]. The significance of immature coagulation and platelet function in the pathophysiology of intravenous hemorrhage is unclear and currently under discussion. Moreover, the definition of coagulopathy in preterm infants is incorrect. Standard coagulation tests, such as the prothrombin time and activated partial thromboplastin time, do not provide a meaningful assessment of hemostatic derangement nor do they give appropriate direction for transfusion management.

Compared to term newborns, moderately preterm infants have lower plasma concentrations of both procoagulant and anticoagulant vitamin K-dependent coagulation factors [37]. However, plasma levels of other proteins and contact pathway components are lowered as well. Current screening tests, such as the PT and APTT, were designed to detect low levels of procoagulant clotting factors but are insensitive to reductions in anticoagulant factors and poor predictors of bleeding or thrombosis in certain high risk clinical settings. The APTT test is sensitive to a decrease in the plasma activity of these components. However, preterm newborns with coexisting abnormalities in anticoagulant pathways may experience a form of hemostatic balance [38]. In addition, the APTT is lengthened by decreases in factor XII and contact pathway factors, none of which are related with a propensity for bleeding or diminished thrombin production. In older preterm babies, the reduction in these contact pathway variables has been clearly described.

The last phase of the coagulation cascade is the stimulation of fibrinogen by thrombin to produce a fibrin plug. Another research evaluated fibrinogen levels between women of the same gestational age who went into preterm labor and those who delivered at term [39]. Due to the considerable rise in fibrinogen levels during the second and third trimesters, which might lead to interpretation error, we matched the weeks of gestation in our investigation. In the other trial, the concentration of fibrinogen was marginally greater in the experimental group than in the control group, but this difference was not statistically significant. In our research, fibrinogen levels were considerably higher in women who gave birth prematurely; however, fibrinogen’s influence was only measured as the fibrinogen to albumin (FAR) score. The FAR score < 0.1 exhibited a sensitivity of 68%, a specificity of 64%, and a 5.30-fold increased risk for preterm delivery.

### 4.2. Study Limitations and Future Perspectives

Beginning with the study’s retroactive approach, this article was limited by several drawbacks. Because the data were obtained from a local hospital, the examined sample may be more homogenous and less applicable to the greater group of pregnant women. Blood specimens were not collected at particular times to avoid certain confounding factors, as one third of all samples in the prematurity group were measured during the early third trimester (weeks 28 to 31), while the other two thirds were measured between 32 and 36 weeks of gestation, based on the moment of birth of each infant. Smoking and being overweight are both known risk factors for preterm birth. While the frequent smoking status and overweight status was significantly more frequent in the preterm birth group, the statistical analysis was adjusted for these confounding factors. Furthermore, we were unable to completely exclude the impact of a particular medication on the observed serum parameters. However, the study concluded with 161 premature newborns and a total size of 322 participants, which provides a sufficient statistical power. Nevertheless, the findings of this study need to be validated in external populations to ensure that the diagnosis methods have a broader applicability. Studies with larger sample sizes and diverse populations are necessary to confirm the accuracy and reliability of these methods.

Regarding the future perspectives and issues of attempting to use these methods in current clinical practice, the cost-effectiveness of using these diagnosis methods in clinical practice should be evaluated. The cost of laboratory testing for these parameters may vary in different healthcare settings, and it is essential to consider the cost-effectiveness of these methods relative to the benefit gained in preventing preterm birth. In addition, the accessibility of these diagnosis methods in low-resource settings may be a challenge. These diagnosis methods require laboratory testing, which may not be easily available in all healthcare settings. Ensuring the availability and accessibility of these methods in all settings is crucial to prevent disparities in preterm birth prevention. The optimal timing of measurement for these diagnosis methods needs to be determined, as in the current study the parameters were measured during the third trimester of pregnancy, but it is unclear whether earlier measurement would provide a better prediction accuracy. Further research is needed to determine the optimal timing of measurement for each parameter. Lastly, it is important to consider the ethical implications of using these diagnosis methods. The diagnosis methods may lead to increased anxiety and stress for women identified as being at high risk of preterm birth. Healthcare providers should ensure that appropriate counseling and support are available to women identified as being at high risk of preterm birth.

## 5. Conclusions

Since prematurity puts the newborn at significantly greater risks, it is important to identify risk factors that are easy to assess. In this study, hemoglobin levels, the association of hemoglobin, albumin, lymphocyte, and platelets’ (HALP) score, and coagulation parameters such as the prothrombin time (PT), activated partial thromboplastin clotting time (aPTT), D-dimers, and fibrinogen to albumin ratio (FAR) during the third trimester of pregnancy, proved to be significant determinants for the risk of preterm birth. However, due to financial constraints, it would be more appropriate to assess these predictors only in pregnancies that are at risk. Nevertheless, further external validation is required to confirm the accuracy of these predictors for premature delivery, since they all showed a poor level of discrimination. It is recommended that these parameters not be applied until more data are presented to support them.

## Figures and Tables

**Table 1:** Background characteristics of the patients analyzed in the study.

Variables	Prematurity (*n* = 161)	No Prematurity (*n* = 161)	*p*-Value
Age (years), mean ± SD	29.4 ± 4.8	29.6 ± 4.9	0.711
Body mass index (kg/m^2^), mean ± SD	26.3 ± 3.5	24.3 ± 3.4	<0.001
Gravidity			0.801
1	107 (66.5%)	103 (64.0%)	
2	34 (21.1%)	39 (24.2%)	
≥3	20 (12.4%)	19 (11.8%)	
Parity			0.505
1	122 (75.8%)	127 (78.9%)	
≥2	39 (24.2%)	34 (21.1%)	
Number of comorbidities *			0.430
0	118 (73.3%)	126 (78.3%)	
1	34 (21.1%)	25 (15.5%)	
≥2	9 (5.6%)	10 (6.2%)	
Substance use during pregnancy			
Smoking	18 (11.2%)	8 (5.0%)	0.040
Alcohol consumption	7 (4.3%)	6 (3.7%)	0.777
Obstetrical characteristics			
Moment of birth (weeks of gestation), mean ± SD	35.3 ± 4.1	37.9 ± 4.8	<0.001
Premature rupture of membranes	17 (10.6%)	4 (2.5%)	0.003
Abnormal placental implantation	16 (9.9%)	9 (5.6%)	0.144
Cesarean delivery	17 (10.6%)	33 (20.5%)	0.013
Urinary tract infections	41 (25.5%)	26 (16.1%)	0.039
Pregnancy loss/miscarriage	6 (3.7%)	4 (2.5%)	0.520
History of abortion	9 (5.6%)	7 (4.3%)	0.608
Distribution of premature births during the third trimester			-
Early third trimester (28–31 weeks)	57 (35.4%)	-	
Late third trimester (32–36 weeks)	104 (64.6%)	-	

Data are presented as *n* (%) and compared with the Chi^2^ test or Fisher’s test unless specified differently; * Comorbidities excluding pregnancy-related complications;

**Table 2:** The analysis of serum parameters is used to assess the risk of premature birth.

Variables	Normal Range *	Prematurity (*n* = 161)	No Prematurity (*n* = 161)	*p*-Value
White blood cell count (×10^9^/L)	4.5–11.0	9.24 ± 5.31	8.23 ± 5.08	0.082
Lymphocytes (×10^9^/L)	1.0–4.8	0.78 ± 0.49	1.06 ± 0.76	<0.001
Platelets (×10^9^/L)	150–450	216 ± 68.4	214 ± 71.0	0.247
Hemoglobin (g/L)	120–160	113 ± 24.7	139 ± 28.5	<0.001
Albumin (g/L)	30–50	31.4 ± 7.0	36.6 ± 6.4	<0.001
PT (sec.)	12.5–13.5	12.9 ± 1.8	13.3 ± 1.7	0.041
aPTT (sec.)	25–35	26.7 ± 2.3	27.9 ± 2.1	<0.001
D-dimers (ng/mL)	<250	229 ± 52	216 ± 36	0.009
Fibrinogen (g/L)	2–4	5.13 ± 0.88	3.10 ± 0.72	<0.001
HALP score	≥24	12.82 ± 3.16	23.96 ± 4.28	<0.001
FAR score	<0.1	0.16 ± 0.02	0.08 ± 0.03	<0.001

Data are presented as mean ± standard deviation and compared using the Student’s *t*-test; PT—prothrombin time; HALP—the association of hemoglobin, albumin, lymphocyte, and platelets’ score; aPTT—activated partial thromboplastin clotting time; FAR—fibrinogen to albumin ratio. * Normal range adjusted for the third trimester of pregnancy.

**Table 3:** ROC plot for the variables involved in estimating the risk for preterm birth.

Variables	AUC	95% CI		SE	%	%	*p*-Value
		Lower Bound	Upper Bound		Sensitivity	Specificity	
Hemoglobin	0.671	0.554	0.819	0.076	73%	65%	0.001
PT	0.625	0.526	0.802	0.073	62%	69%	0.042
aPTT	0.632	0.561	0.886	0.070	68%	64%	0.045
D-dimers	0.615	0.487	0.731	0.071	66%	60%	0.039
HALP	0.680	0.548	0.835	0.084	75%	69%	0.001
FAR	0.646	0.533	0.774	0.080	68%	64%	0.020

AUC—Area Under Curve; CI—Confidence Interval; SE—Standard Error; PT—prothrombin time; HALP—the association of hemoglobin, albumin, lymphocyte, and platelets’ score; aPTT—activated partial thromboplastin clotting time; FAR—fibrinogen to albumin ratio.

**Table 4:** Regression analysis.

Adjusted Factors	Odds Ratio	(95% CI)	*p*-Value
Hemoglobin	3.28	1.62–5.36	0.001
PT	2.11	1.09–4.91	0.038
aPTT	3.24	2.01–6.13	<0.001
D-dimers	4.26	1.24–6.58	0.005
HALP	6.09	1.99–10.24	<0.001
FAR	5.30	1.75–8.87	<0.001

CI—Confidence Interval; PT—prothrombin time; HALP—the association of hemoglobin, albumin, lymphocyte, and platelets’ score; aPTT—activated partial thromboplastin clotting time; FAR—fibrinogen to albumin ratio; the statistical analysis was adjusted for confounding factors (age, weight, and smoking status).

## Data Availability

The data presented in this study are available on request from the corresponding author.

## References

[1] Samuel T.M., Sakwinska O., Makinen K., Burdge G.C., Godfrey K.M., Silva-Zolezzi I. (2019). Preterm Birth: A Narrative Review of the Current Evidence on Nutritional and Bioactive Solutions for Risk Reduction. Nutrients.

[2] Romero R., Dey S.K., Fisher S.J. (2014). Preterm labor: One syndrome, many causes. Science.

[3] Liu L., Oza S., Hogan D., Perin J., Rudan I., Lawn J.E., Cousens S., Mathers C., Black R.E. (2015). Global, regional, and national causes of child mortality in 2000–13, with projections to inform post-2015 priorities: An updated systematic analysis. Lancet.

[4] Kugelman A., Colin A.A. (2013). Late Preterm Infants: Near Term but Still in a Critical Developmental Time Period. Pediatrics.

[5] Georgiou H.M., Di Quinzio M.K.W., Permezel M., Brennecke S.P. (2015). Predicting Preterm Labour: Current Status and Future Prospects. Dis. Markers.

[6] Kajantie E., Strang-Karlsson S., Evensen K.A.I., Haaramo P. (2019). Adult outcomes of being born late preterm or early term–what do we know?. Semin. Fetal Neonatal Med..

[7] Halimi Asl A.A., Safari S., Parvareshi Hamrah M. (2017). Epidemiology and Related Risk Factors of Preterm Labor as an obstetrics emergency. Emergency.

[8] Dahman H. (2020). Risk factors associated with preterm birth: A retrospective study in Mukalla Maternity and Childhood Hospital, Hadhramout Coast/Yemen. Sudan. J. Paediatr..

[9] Alijahan R., Hazrati S., Mirzarahimi M., Pourfarzi F., Hadi P.A. (2014). Prevalence and risk factors associated with preterm birth in Ardabil, Iran. Iran. J. Reprod. Med..

[10] Kemp M., Newnham J., Challis J., Jobe A., Stock S. (2015). The clinical use of corticosteroids in pregnancy. Hum. Reprod. Updat..

[11] Ma M., Zhu M., Zhuo B., Li L., Chen H., Xu L., Wu Z., Cheng F., Xu L., Yan J. (2020). Use of complete blood count for predicting preterm birth in asymptomatic pregnant women: A propensity score-matched analysis. J. Clin. Lab. Anal..

[12] Berger R., Rath W., Abele H., Garnier Y., Kuon R.-J., Maul H. (2019). Reducing the Risk of Preterm Birth by Ambulatory Risk Factor Management. Dtsch. Ärzteblatt Int..

[13] Vinall J., Miller S.P., Bjornson B.H., Fitzpatrick K.P., Poskitt K.J., Brant R., Synnes A.R., Cepeda I.L., Grunau R.E. (2014). Invasive Procedures in Preterm Children: Brain and Cognitive Development at School Age. Pediatrics.

[14] Zhang Q., Ananth C.V., Li Z., Smulian J.C. (2009). Maternal anaemia and preterm birth: A prospective cohort study. Leuk. Res..

[15] Wasim T., Bushra N., Tajammul A., Humayun S., Rasool S., Shahbaz F., Riaz A., Siddique F., Khawaja K.I., Fatima A. (2023). Ferritin screening and Iron treatment for maternal anemia and fetal growth restriction prevention—A multicenter randomized controlled trial (FAIR Study). Pak. J. Med. Sci..

[16] Hrubaru I., Motoc A., Moise M.L., Miutescu B., Citu I.M., Pingilati R.A., Popescu D.-E., Dumitru C., Gorun F., Olaru F. (2022). The Predictive Role of Maternal Biological Markers and Inflammatory Scores NLR, PLR, MLR, SII, and SIRI for the Risk of Preterm Delivery. J. Clin. Med..

[17] Quinn J.-A., Munoz F.M., Gonik B., Frau L., Cutland C., Mallett-Moore T., Kissou A., Wittke F., Das M., Nunes T. (2016). Preterm birth: Case definition & guidelines for data collection, analysis, and presentation of immunisation safety data. Vaccine.

[18] Timircan M., Bratosin F., Vidican I., Suciu O., Turaiche M., Bota A.V., Mitrescu S., Marincu I. (2021). Coping Strategies and Health-Related Quality of Life in Pregnant Women with SARS-CoV-2 Infection. Medicina.

[19] Xi F., Chen H., Chen Q., Chen D., Chen Y., Sagnelli M., Chen G., Zhao B., Luo Q. (2021). Second-trimester and third-trimester maternal lipid profiles significantly correlated to LGA and macrosomia. Arch. Gynecol. Obstet..

[20] Shen X.-B., Zhang Y.-X., Wang W., Pan Y.-Y. (2019). The Hemoglobin, Albumin, Lymphocyte, and Platelet (HALP) Score in Patients with Small Cell Lung Cancer Before First-Line Treatment with Etoposide and Progression-Free Survival. Experiment.

[21] Leetanaporn K., Hanprasertpong J. (2022). Predictive Value of the Hemoglobin-Albumin-Lymphocyte-Platelet (HALP) Index on the Oncological Outcomes of Locally Advanced Cervical Cancer Patients. Cancer Manag. Res..

[22] Cong L., Hu L. (2017). The value of the combination of hemoglobin, albumin, lymphocyte and platelet in predicting platinum-based chemoradiotherapy response in male patients with esophageal squamous cell carcinoma. Int. Immunopharmacol..

[23] Peng D., Zhang C.-J., Tang Q., Zhang L., Yang K.-W., Yu X.-T., Gong Y., Li X.-S., He Z.-S., Zhou L.-Q. (2018). Prognostic significance of the combination of preoperative hemoglobin and albumin levels and lymphocyte and platelet counts (HALP) in patients with renal cell carcinoma after nephrectomy. BMC Urol..

[24] Seong W.J., Chong G.O., Hong D.G., Lee T.H., Lee Y.S., Cho Y.L., Chun S.S., Park I.S. (2010). Clinical significance of serum albumin level in pregnancy-related hypertension. J. Obstet. Gynaecol. Res..

[25] Saitou T., Watanabe K., Kinoshita H., Iwasaki A., Owaki Y., Matsushita H., Wakatsuki A. (2021). Hypoalbuminemia is related to endothelial dysfunction resulting from oxidative stress in parturients with preeclampsia. Nagoya J. Med. Sci..

[26] Rahmati S., Azami M., Badfar G., Parizad N., Sayehmiri K. (2019). The relationship between maternal anemia during pregnancy with preterm birth: A systematic review and meta-analysis. J. Matern. Neonatal Med..

[27] Ardic C., Usta O., Omar E., Yıldız C., Memis E., Öztürk G.Z. (2019). Relationship between anaemia during pregnancy and preterm delivery. J. Obstet. Gynaecol..

[28] Sunuwar D.R., Singh D.R., Chaudhary N.K., Pradhan P.M.S., Rai P., Tiwari K. (2020). Prevalence and factors associated with anemia among women of reproductive age in seven South and Southeast Asian countries: Evidence from nationally representative surveys. PLoS ONE.

[29] Opitasari C., Andayasari L. (2015). Young Mothers, Parity and the Risks of Anemia in the Third Trimester of Pregnancy. Health Sci. J. Indones..

[30] Owais A.M., Umay K. (2015). Effect of maternal anaemia on APGAR score of Newborn. J. Rawalpindi Med. Coll. (JRMC).

[31] Chaudhary N., Yadav Shree N., Kalra Suresh K., Pathak S., Gupta Binod K., Shrestha S., Patel M., Satia I., Sadhra S., Bolton Charlotte E. (2021). Prognostic Factors Associated with Small for Gestational Age Babies in a Tertiary Care Hospital of Western Nepal: A Cross-sectional Study. Health Sci. Rep..

[32] Badfar G., Shohani M., Soleymani A., Azami M. (2018). Maternal anemia during pregnancy and small for gestational age: A systematic review and meta-analysis. J. Matern. Neonatal Med..

[33] Ivan E.A., Mangaiarkkarasi A. (2013). Evaluation of anaemia in booked antenatal mothers during the last trimester. J. Clin. Diagn. Res..

[34] Suryanarayana R., Chandrappa M., Santhuram A.N., Prathima S., Sheela S. (2017). Prospective study on prevalence of anemia of pregnant womenand its outcome: A community based study. J. Fam. Med. Prim. Care.

[35] Keren-Politansky A., Breizman T., Brenner B., Sarig G., Drugan A. (2014). The coagulation profile of preterm delivery. Thromb. Res..

[36] Hochart A., Nuytten A., Pierache A., Bauters A., Rauch A., Wibaut B., Susen S., Goudemand J. (2019). Hemostatic profile of infants with spontaneous prematurity: Can we predict intraventricular hemorrhage development?. Ital. J. Pediatr..

[37] Neary E., McCallion N., Kevane B., Cotter M., Egan K., Regan I., Kirkham C., Mooney C., Coulter-Smith S., Áinle F.N. (2015). Coagulation indices in very preterm infants from cord blood and postnatal samples. J. Thromb. Haemost..

[38] Monagle P., Massicotte P. (2011). Developmental haemostasis: Secondary haemostasis. Semin, Fetal Neonatal Med..

[39] Hansen A.T., Andreasen B.H., Salvig J.D., Hvas A.-M. (2010). Changes in fibrin D-dimer, fibrinogen, and protein S during pregnancy. Scand. J. Clin. Lab. Investig..

